# Ivermectin effectively inhibits hepatitis E virus replication, requiring the host nuclear transport protein importin α1

**DOI:** 10.1007/s00705-021-05096-w

**Published:** 2021-05-14

**Authors:** Yunlong Li, Zhijiang Miao, Pengfei Li, Ruyi Zhang, Denis E. Kainov, Zhongren Ma, Robert A. de Man, Maikel P. Peppelenbosch, Qiuwei Pan

**Affiliations:** 1 Department of Gastroenterology and Hepatology, Erasmus MC-University Medical Center, Room Na-1005, Wytemaweg 80, 3015 CN Rotterdam, The Netherlands; 2 Department of Clinical and Molecular Medicine, Norwegian University of Science and Technology, 7028 Trondheim, Norway; 3 Institute of Technology, University of Tartu, 50090 Tartu, Estonia; 4 Biomedical Research Center, Northwest Minzu University, Lanzhou, China

## Abstract

We show that ivermectin, an FDA-approved anti-parasitic drug, effectively inhibits infection with hepatitis E virus (HEV) genotypes 1 and 3 in a range of cell culture models, including hepatic and extrahepatic cells. Long-term treatment showed no clear evidence of the development of drug resistance. Gene silencing of importin-α1, a cellular target of ivermectin and a key member of the host nuclear transport complex, inhibited viral replication and largely abolished the anti-HEV effect of ivermectin.

**Supplementary Information:**

The online version contains supplementary material available at 10.1007/s00705-021-05096-w.

Hepatitis E virus (HEV), a single-stranded, positive-sense RNA virus, is the most common cause of acute viral hepatitis worldwide [1]. Of the eight classified genotypes, genotypes 1, 2, 3, and 4 are known to infect humans [2]. In general, HEV infection is self-limiting, with an extremely low mortality rate. However, pregnant women acutely infected with HEV genotype 1 have a high risk of developing severe complications, with a mortality rate of up to 30% [3]. Organ transplantation recipients infected with genotype 3, and occasionally genotype 4, have a high risk of developing chronic hepatitis E [4–6]. Because no FDA-approved medication is available, therapy with interferon alpha (IFNα), ribavirin, or their combination has been used as an off-label treatment for chronic hepatitis E [7, 8]. However, the use of IFNα is generally contraindicated in kidney, pancreas, heart, and lung-transplant recipients because it stimulates the immune system and increases the risk of acute rejection [9]. Ribavirin monotherapy is effective in approximately 80% of chronic hepatitis E patients who are eligible for treatment [10]. However, treatment failure has been reported frequently, probably due to development of resistance or poor tolerance [7]. Thus, there is a clinical need to develop new antiviral therapies against HEV, and we are interested in repurposing safe-in-humans broad-spectrum antiviral drugs for treating hepatitis E [11].

Ivermectin is an approved anti-parasitic medication with an established safety profile since the 1970s, and it is on the World Health Organization (WHO) list of essential medicines [12, 13]. Over the last 30 years, there were more than 2.5 billion doses of ivermectin distributed across the globe, showing an excellent safety profile in the clinic [14]. Interestingly, ivermectin has been reported to exert broad antiviral activity against single-stranded RNA viruses and DNA viruses. More interestingly, ivermectin has recently been shown to inhibit the replication of SARS-CoV-2, the causative agent of the COVID-19 pandemic [15]. In this study, we evaluated the ability of ivermectin to inhibit HEV replication and investigated its possible mechanism of action.

To test the potential anti-HEV activity of ivermectin, we used a subgenomic replicon and a full-length HEV infectious clone, based on the genotype 3 Kernow-C1 p6 clone as models (Supplementary Fig. S1). In the subgenomic model, open reading frame (ORF) 2, encoding the capsid protein, was replaced by a *Gaussia* luciferase reporter gene for monitoring viral replication. We found that ivermectin treatment significantly inhibited viral-replication-dependent luciferase activity in human hepatic Huh7 cells harboring the subgenomic replicon in a dose-dependent manner (Fig. 1A). We next tested a series of ivermectin concentrations (0.001-100 μM) in the Huh7 cell model to assess antiviral and cytotoxic effects. The 50% inhibitory and cytotoxic (IC_50_ and CC_50_) concentrations of ivermectin were 2.68 μM and 18.83 μM, respectively (Fig. 1B). In the Huh7 infectious p6 cell model, treatment with ivermectin for 48 hours potently inhibited HEV viral RNA replication, as determined by qRT-PCR (Fig. 1C and Supplementary Fig. S2A), and HEV ORF2 protein expression, as measured by western blotting (Fig. 1D). Treatment with 10 μM ivermectin for 48 hours resulted in a 71.68% ± 5.78 (mean ± SEM, n = 4, *p* < 0.001) (Fig. 1C) and 51.67% ± 8.19 (mean ± SEM, n = 4, *p* < 0.01) (Fig. 1D) reduction in the HEV RNA and capsid protein level, respectively. Furthermore, based on calculation of the viral RNA copy number (Supplementary Fig. S2B), treatment with 1 or 5 μM ivermectin for 48 hours resulted in significant inhibition of secreted viral RNA into the supernatant (Supplementary Fig. S2C), which was consistent with the reduced level of capsid protein in the supernatant (Supplementary Fig. S2D). The inhibitory effect was confirmed using a confocal imaging assay, which showed a dose-dependent decrease in ORF2-encoded capsid protein expression (Fig. 1E). Furthermore, ivermectin also inhibited replication of a Sar55 clone-based genotype 1 HEV subgenomic replicon in a dose-dependent manner (Fig. 1F). Since the p6 genotype 3 clone is capable of producing infectious viral particles, we harvested HEV particles 48 hours after treatment with the antiviral drug and then performed a re-infection assay in naïve Huh7 cells to evaluate the antiviral effects. Ribavirin was included as a positive control. We found that the amount of infectious HEV produced was significantly reduced by ivermectin or ribavirin treatment, as shown by decreases in both the viral RNA and protein levels upon re-infection of Huh7 cells (Supplementary Fig. S3). Notably, HEV was found to be more sensitive to ivermectin than to ribavirin. For example, 10 μM ivermectin exerted potent anti-HEV activity, whereas the antiviral activity of ribavirin at this concentration was very low (Supplementary Fig. S3). Drug resistance is one of the main factors that limit the effectiveness of antiviral treatment. Long-term exposure to low-dose antiviral drugs can lead to the development of resistance [16]. To characterize ivermectin in this respect, the HEV p6 cell model was continuously exposed to a low dose of ivermectin (1 μM). Ivermectin retained its anti-HEV activity even after 21 days, causing a reduction in viral RNA and capsid protein levels (Fig. 1G).

**Fig. 1:**
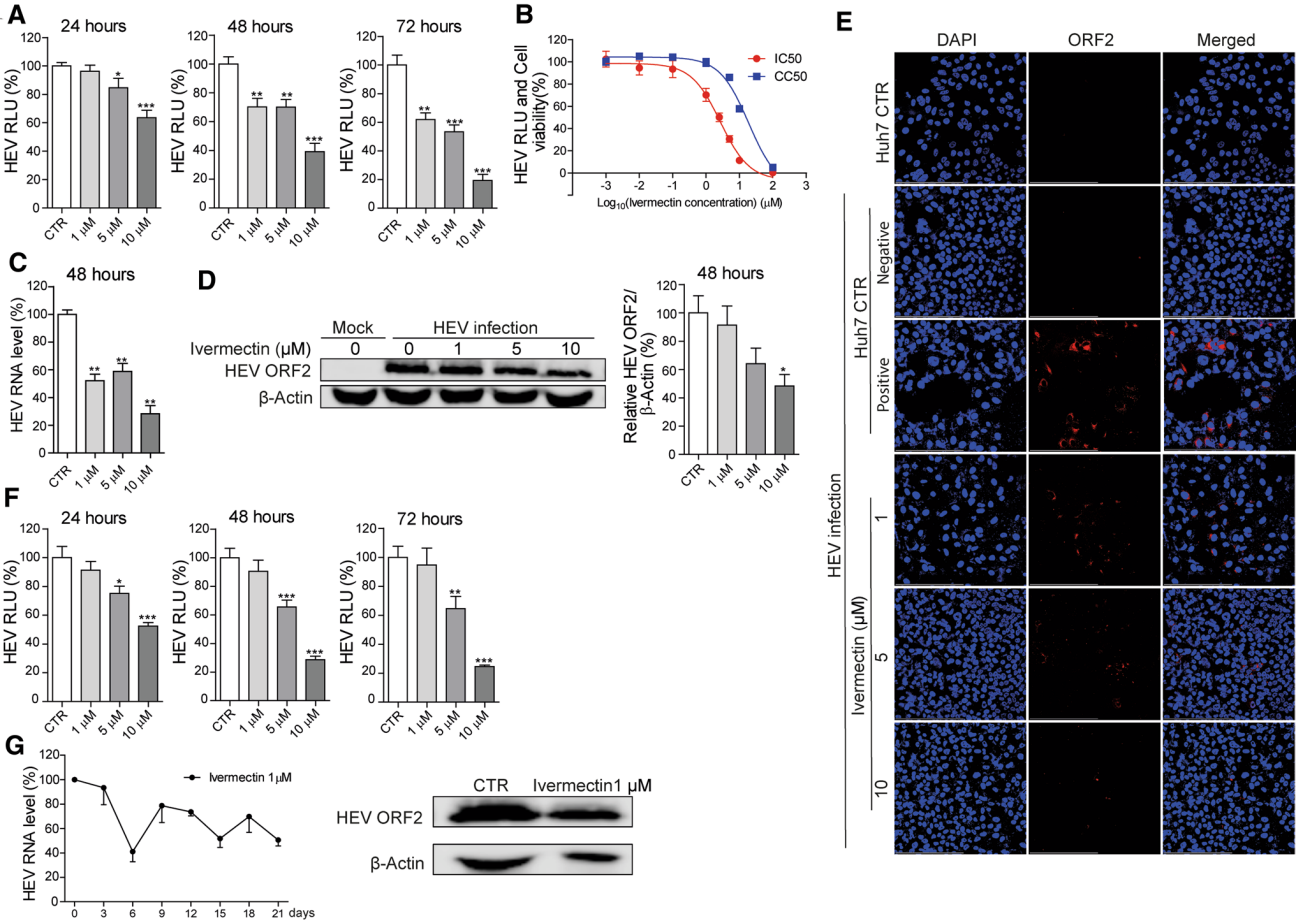
Anti-HEV activity of ivermectin in Huh7-based cell culture models. (A) Effect of ivermectin treatment for 24, 48, or 72 hours on viral replication-dependent luciferase activity in the genotype 3 subgenomic Huh7-p6-Luc cell model. The untreated group served as a control (CTR) (set as 100%) (n = 12). (B) The 50% inhibitory concentration (IC_50_) and 50% cytotoxic concentration (CC_50_) of ivermectin in Huh7-p6-Luc cell model and Huh7 cell line were calculated using GraphPad Prism 5 software (n = 6-12). (C) The infectious Huh7-p6 cell model was treated with the indicated concentrations of ivermectin for 48 hours. The effect on viral RNA production was quantified by qRT-PCR using primers targeting the ORF2/ORF3 overlap region (n = 4-8). (D) Western blot analysis of the HEV capsid protein level in Huh7-p6 cells treated with ivermectin for 48 hours. The uninfected group (mock) served as a negative control, and the infected but untreated group served as a positive control (set as 100%) (n = 4). (E) Immunofluorescence analysis of viral ORF2-encoded capsid protein (red) in Huh7 cells treated with the indicated concentrations of ivermectin for 48 hours. Huh7 cells incubated with an anti-HEV capsid protein antibody or Huh7-p6 cells incubated with the matched IgG control antibody served as a negative control. Untreated HEV-infected Huh7 cells incubated with the anti-HEV capsid protein antibody served as a positive control. DAPI (blue) was applied to visualize nuclei (40 × oil immersion objective). (F) Huh7 cells containing the genotype 1 HEV replicon (Sar55 clone) were treated with ivermectin for 24, 48, or 72 hours, and viral-replication-dependent luciferase activity was measured (n = 10). (G) The effects of long-term treatment with 1 μM ivermectin on HEV RNA replication in the Huh7-p6 cell model were analyzed by qRT-PCR. The untreated (CTR) group served as a control (set as 100%) (n = 3-4). Western blot of the HEV ORF2-encoded capsid protein performed after 21 days of treatment. RLU, relative luciferase units. Data are presented as the mean ± SEM (*, *P* < 0.05; **, *P* < 0.01; ***, *P* < 0.001).

In addition to hepatitis, HEV infection is associated with a broad range of extrahepatic manifestations, including kidney damage and neurological diseases [17–19]. We therefore tested the antiviral activity of ivermectin in a variety of cell models, including hepatic and non-hepatic cell lines, using HEV genotype 3. Notably, ivermectin significantly inhibited viral-replication-dependent luciferase activity in human kidney 293T (Fig. 2A), hepatic PLC (Fig. 2B), and neuronal U87 (Fig. 2C) cells. The IC_50_ and CC_50_ values were 2.89 μM and 18.23 μM, respectively, in the 293T cell model, 3.75 μM and 68.78 μM in the PLC cell model, and 7.83 μM and 49.51 μM in U87 cell model.

**Fig. 2:**
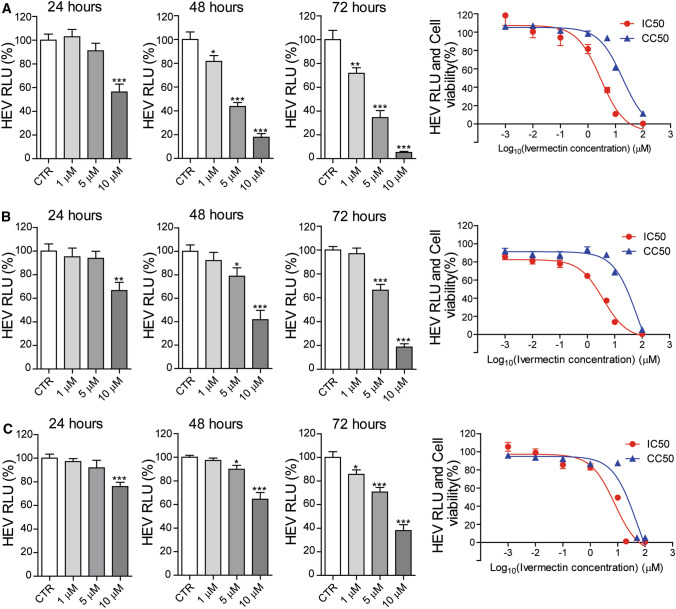
Anti-HEV activity of ivermectin in different human cell culture models. The effects of ivermectin treatment on viral-replication-dependent luciferase activity, the 50% inhibitory concentration (IC_50_) against HEV replication, and the 50% cytotoxic concentration (CC_50_) of ivermectin in different cells were calculated using GraphPad Prism 5 software. (A) 293T kidney cells (n = 6-12) (B) PLC/PRF/5 cells (n = 10-12) (C) U87 neuronal cells (n = 10-12). The untreated (CTR) group served as control (set as 100%). RLU: relative luciferase units. Data are presented as the mean ± SEM (*, *P* < 0.05; **, *P* < 0.01; ***, *P* < 0.001).

There is emerging evidence that the antiviral activity of ivermectin against particular viruses is associated with the host nuclear transport protein importin (IMP) α/β, which may be attributed to the inhibition of nuclear import of host and viral proteins [20–22]. For example, the antiviral activity of ivermectin against human immunodeficiency virus 1 (HIV-1) and dengue virus has been attributed to the inhibition of IMP α/β nuclear import [21]. A very recent study has demonstrated that ivermectin can dissociate the preformed IMP α/β heterodimer and prevent complex formation to limit West Nile virus infection [22]. Although IMPα and IMPβ are commonly known to form a heterodimer complex, they are actually represented by a large family of proteins encoded by various genes. In humans, IMPα includes α1, α3, α4, α5, α6, α7, and α8, which are encoded by the KPNA2, KPNA4, KPNA3, KPNA1, KPNA5, KPNA6, and KPNA7 genes, respectively [23]. Among these genes, KPNA2, which encodes IMPα1 in humans and IMPα2 in mice, has been implicated in mediating the antiviral activity of ivermectin against West Nile virus infection [22]. Thus, we further investigated the role of the human KPNA2 gene and the IMPα1 protein in the setting of an HEV infection. We used lentiviral shRNA vectors to stably knock down the KPNA2 gene in Huh7 cells and confirmed the gene silencing efficacy at both the mRNA (Fig. 3A) and protein (Fig. 3B) levels, as compared to a control. Two clones with optimal gene knockdown levels were selected for subsequent functional experimentation. Compared to the control, knockdown of KPNA2 by shKPNA2 clones 2 and 3 led to 73% ± 4.6 (mean ± SEM, n = 4, *p* < 0.05) and 64% ± 13 (mean ± SEM, n = 4, *p* < 0.05) reduction, respectively, in the intracellular HEV RNA level (Fig. 3C), suggesting that IMPα1 is a host factor that supports HEV infection. Importantly, the anti-HEV activity of ivermectin was largely abolished in KPNA2-knockdown cells, demonstrating that IMPα1 is required for the anti-HEV action of ivermectin (Fig. 3D). To assess the specificity of KPNA2/IMPα1, we performed a similar gene knockdown of KPNA1 (Supplementary Fig. S4A), which encodes IMPα5 in humans. In contrast, the loss of IMPα5 had no effect on HEV replication (Supplementary Fig. S4B) and did not reverse the anti-HEV activity of ivermectin (Supplementary Fig. S4C). Thus, we have demonstrated that a component of the nuclear transport machinery, IMPα1, mediates the anti-HEV activity of ivermectin. However, the mechanism by which the IMP α/β heterodimer nuclear import complex is involved in HEV infection and mediates the anti-HEV activity of ivermectin requires further study.

**Fig. 3:**
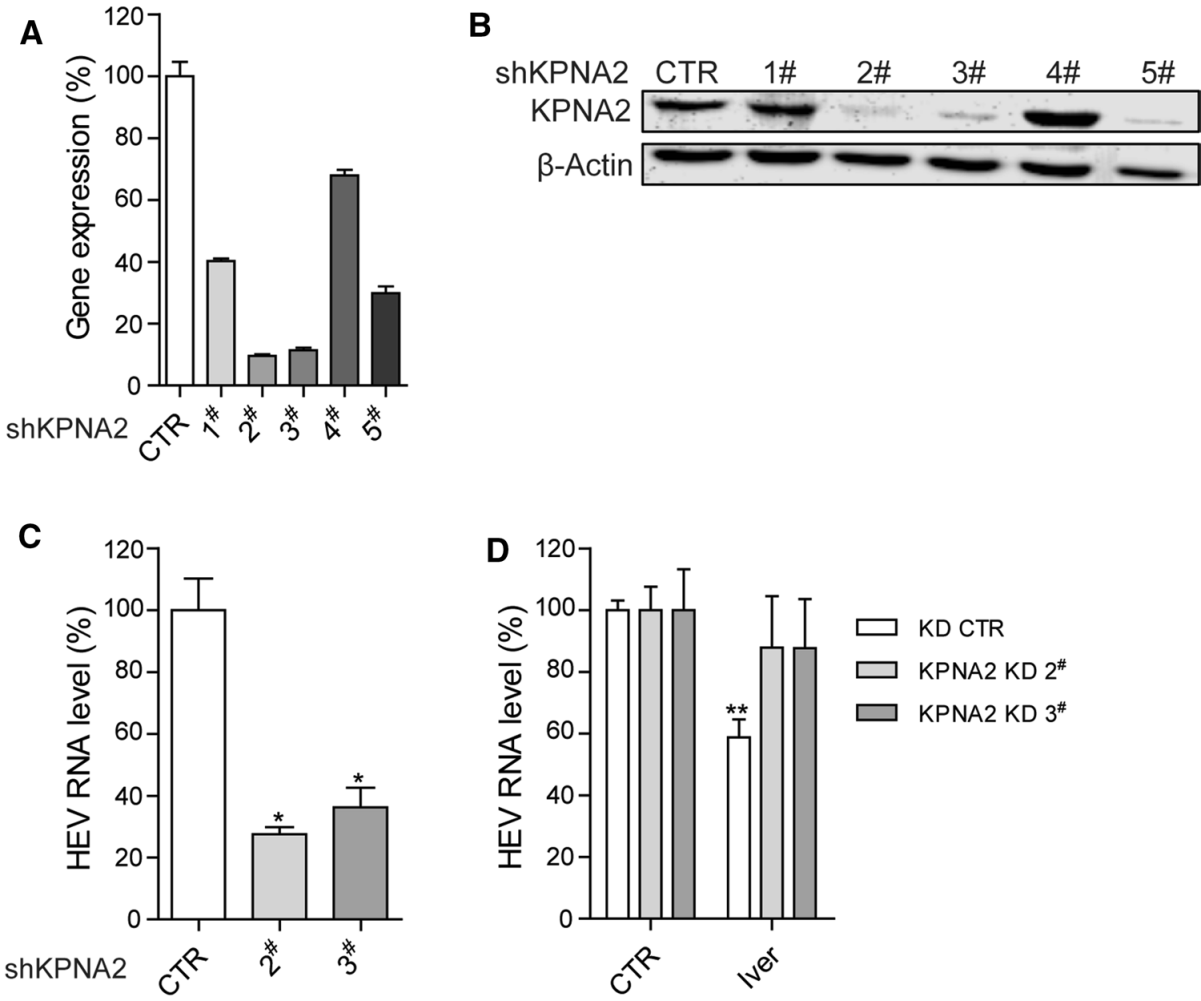
The effect of KPNA2 gene silencing on HEV replication and the anti-HEV activity of ivermectin. (A) Knockdown of KPNA2 by lentiviral shRNA vectors. The effect on the KPNA2 gene of five shKPNA2 clones was quantified by qRT-PCR (n = 4). The expression of KPNA2 in control-vector-transduced cells was set as 100%. (B) Western blot analysis of the KPNA2-encoded IMPα1 protein level. (C) The effect of KPNA2 knockdown on cellular HEV RNA levels in the Huh7 p6 cell model. Viral RNA was quantified by qRT-PCR (n = 4). (D) KPNA2 knockdown and control Huh7 cells harboring the infectious HEV model were treated with 5 μM ivermectin for 48 hours. The effect on viral RNA production was quantified by qRT-PCR (n = 6-8). Data are presented as the mean ± SEM (*, *P* < 0.05; **, *P* < 0.01; ***, *P* < 0.001).

Combining multiple antiviral drugs is a common strategy to enhance treatment efficacy and prevent development of resistance. Since IFNα, ribavirin, and mycophenolic acid (MPA) have shown anti-HEV activity in cell culture systems [24, 25], combining ivermectin with these compounds can thus be envisaged. Surprisingly, however, combination with ivermectin antagonized the anti-HEV effects of IFNα (Supplementary Fig. S5A), ribavirin (Supplementary Fig. S5B), and MPA (Supplementary Fig. S5C).

In summary, this study comprehensively demonstrates the anti-HEV activity of ivermectin and a potential mechanism involving the host factor IMPα1. Our findings support further exploration of the potential for repurposing ivermectin for treating HEV infection, including for organ transplantation recipients with chronic hepatitis E. Parasitic infections are important complications of organ transplantation that may cause serious morbidity if overlooked. Ivermectin treatment is an effective and commonly used therapy in strongyloid-seropositive organ transplant recipients [26, 27]. Although ivermectin use may occasionally be associated with very mild and self-limiting liver damage, it has been used for treating hyperinfection strongyloidiasis in liver transplant patients with satisfactory outcomes [28, 29]. Thus, ivermectin is uniquely suitable for treating patients with parasite and HEV co-infections, but it also has potential for treating HEV infection in general. Of note, our results are mainly based on the cell-culture-adapted genotype 3 Kernow-C1 p6 HEV strain, which may differ from clinical strains. Nevertheless, our findings warrant further studies to evaluate the efficacy and safety profiles of prolonged ivermectin treatment for treating HEV infection, preferably in animal models first, before moving into human clinical trials.

## Materials and methods

In this study, the detailed Materials and methods are provided as a supplementary file.

## Supplementary Information

Below is the link to the electronic supplementary material.

Supplementary file1 (DOCX 1507 kb)

## Declarations

### Research involving human and animal participants

This article does not contain any studies with human participants or animals performed by any of the authors and is in compliance with ethical standards for research.
