# Supplementary material for: Ivermectin effectively inhibits hepatitis E virus replication, requiring the host nuclear transport protein importin α1
Source: Arch Virol. 2021 May 14;166(7):2005–10. doi: 10.1007/s00705-021-05096-w (PMC8119233; doi:10.1007/s00705-021-05096-w)
Supplement: Supplementary file 1 — Supplementary file1 (DOCX 1507 kb) [file 705_2021_5096_MOESM1_ESM.docx]

**Ivermectin effectively inhibits hepatitis E virus replication requiring the host nuclear transport importin α1**

Yunlong Li, Zhijiang Miao, Pengfei Li, Ruyi Zhang, Denis E. Kainov, Zhongren Ma, Robert A. de Man, Maikel P. Peppelenbosch, Qiuwei Pan*

**Materials and Methods**

**Reagents and antibodies.** Ivermectin was purchased from Sigma-Aldrich and dissolved in Dimethyl sulfoxide (DMSO, Sigma, Zwijndrecht, the Netherlands) with stock concentration of 100 mM. Human IFNα, ribavirin and mycophenolic acid (MPA) were purchased from Sigma-Aldrich (Zwijndrecht, The Netherlands). The anti-HEV capsid protein antibody was purchased from EMD Millipore (mouse monoclonal, MAB8002). Mouse Control IgG2b antibody was purchased from InvivoGen (mabg2b-ctrlm). Anti-KPNA2 antibody (rabbit, #14372) was obtained from Cell Signaling Technology. Anti-β-actin antibody (mouse monoclonal, sc-47778) was obtained from Santa Cruz Biotechnology (Santa Cruz, CA, USA).

**Cell culture.** Human hepatoma Huh7 and PLC/PRF/5 (PLC) cell lines, human embryonic kidney epithelial cell line (293T), human glioblastoma cell line (U87) were kindly provided by the Department of Viroscience (Erasmus Medical Center) and cultured in Dulbecco’s modified Eagle medium (DMEM) supplemented with 10% fetal bovine serum, 100 IU/mL penicillin, and 100 IU/mL streptomycin.

**Viruses and cell culture models.** Genotype 3 HEV models are based on a plasmid construct containing the full-length HEV genome (genotype 3 Kernow-C1 p6 clone, GenBank accession number JQ679013) or a construct containing subgenomic HEV sequence in which ORF2 was replaced by a Gaussia luciferase reporter gene (p6-Luc). Viral RNA was produced by using the Ambion mMESSAGE nMACHINE in vitro RNA transcription kit. Cells were electroporated with p6 full-length HEV RNA or p6-Luc subgenomic RNA to generate infectious or luciferase-based replicon models, respectively. Similarly, genotype 1 replicon model is based on the Sar 55/S17/luc (GenBank accession number AF444002) HEV clone containing a Gaussia luciferase reporter [1].

**MTT assay.** 10 mM 3-(4,5-dimethylthiazol-2-yl)-2,5-diphenyltetrazolium bromide (MTT) (Sigma) was added to the cells seeded in 96-well plate and cells were maintained at 37℃ with 5% CO_2_ for 3 hours. Medium was removed and 100 μL of DMSO was added to each well. The absorbance of each well was read on the microplate absorbance readers (BIO-RAD) at wavelength of 490 nm.

**Quantification of viral replication.** Viral replication in HEV replication models was monitored by the activity of secreted Gaussia luciferase measured by QUANTI-Luc™ Gold (InvivoGen). Luciferase activity was quantified with a LumiStar Optima luminescence counter (BMG Lab Tech, Offenburg, Germany). For HEV infectious model, viral RNA was quantified. RNA was isolated using a Machery-Nucleo Spin RNA II kit (Bioke, Leiden, The Netherlands) and quantified using a NanoDrop ND-1000 spectrophotometer (Wilmington, DE, USA). cDNA was prepared from total RNA using a cDNA Synthesis Kit (Takara Bio Inc, USA). The HEV RNA level was quantified using a SYBR Green–based real-time PCR assay (Applied Biosystems SYBR Green PCR Master Mix, Life Technologies, CA, USA) according to the manufacturer’s instructions. Glyceraldehyde-3-Phosphate Dehydrogenase (GAPDH) was used as housekeeping gene to normalize gene expression using the 2-∆∆Ct method. The primer sequences were as follows: HEV ORF2/ORF3 overlap region sense, 5’-GGTGGTTTCTGGGGTGAC-3’; HEV ORF2/ORF3 overlap region anti sense, 5’- AGGGGTTGGTTGGATGAA -3’; HEV RdRp region sense, 5’- TGAGGAGTCAGTGCTTGCTG - 3’; HEV RdRp region antisense, 5’- ATGCCGCACTCCTCCATAAC -3’; GAPDH sense, 5’- GTCTCCTCTGACTTCAACAGCG -3’; GAPDH anti sense, 5’- ACCACCCTGTTGCTGTAGCCAA-3’.

**Western Blot.** Proteins in cell lysates were heated at 95°C for 5 min, followed by loading onto a 10% sodium dodecyl sulfate polyacrylamide gel (SDS-PAGE), separated at 90 V for 120 min, and electrophoretically transferred onto a polyvinylidene difluoride (PVDF) membrane (pore size: 0.45 mm; Thermo Fisher Scientific Life Sciences) for 120 min with an electric current of 250 mA. Subsequently, the membrane was blocked with blocking buffer (Li-Cor Biosciences) for 1 hour at room temperature. Membrane was followed by incubation with primary antibodies mouse anti-HEV ORF2 (1:1000) and anti-β-actin (1:1000) overnight at 4°C. The membrane was washed 3 times followed by incubation for 1 hour with anti-mouse IRDye-conjugated secondary antibodies (1:5000; Li-Cor Biosciences) at room temperature. After washing 3 times, protein bands were detected with Odyssey 3.0 Infrared Imaging System.

**Virus production and re-infection assay.** Huh7 cells harboring the infectious genotype 3 HEV were seeded into a multi - well plates HEV particles were harvested by repeated freezing and thawing 3 times, and filtered by 0.45 μm filters. Naïve Huh7 cells were seeded into a multi-well plate and culture medium was discarded when cell confluence was approximately 80%, followed by twice 1×PBS washing. Harvested viruses were added and incubated at 37 ̊C with 5% CO_2_ for 24 hours for re-infection, followed by 3 times washing with 1× PBS to remove unattached viruses. Then cells were incubated with culture medium for another 48 hours. The infectivity of produced HEV particles were analyzed by qRT-PCR and Western blotting, respectively.

**Long-term treatment assay.** For the long-term treatment assay of infectious model (Huh7-p6), the cells were seeded into a 48-well plate with 2 × 10^4^ cells per well. The cells of the control (CTR) or ivermectin treatment groups were passaged and seeded with the same number of cells every 3 days, and cells incubated with normal or contained ivermectin (1 μM) medium were maintained throughout the entire incubation period.

**Immunofluorescence.** Cells grown on µ-Slide 8 Well (ibidi GmbH, 80826) were fixed with 4% (w/v) paraformaldehyde (PFA) for 10 min at room temperature. After three washes with PBS buffer, cells were permeabilized with 0.1% (v/v) Triton X-100 for 10 min and washed three times with PBS. Block for 1h at Room Temperature with blocking solution (5% Normal Donkey Serum, 1% Bovine Serum Albumin, 0.2% TRITON X in 1 × PBS). Cells were then incubated with primary anti-HEV capsid protein (1:200) antibody (aa 434-457, clone 1E6, IgG2b) or matched mouse IgG 2b antibody (1:200) (InvivoGen) at 4°C overnight and washed three times with PBS, followed by incubation with Anti-mouse-Alexa Fluor® 594-Conjugate antibody (Cell Signaling Technology) (1:5000) for 1 hour at room temperature. Nucleus was stained with 4,6-diamidino-2-phenylindole (DAPI) for 10 min at room temperature. Images were detected with confocal electroscope (lens: 40×).

**Gene knockdown by lentiviral vectors.** Lentiviral pLKO knockdown vectors (Sigma-Aldrich) expressing shRNAs targeting KPNA1 or KPNA2 and their appropriate controls were obtained from the Erasmus Biomics Center and were produced in HEK 293T cells. These shRNA sequences are listed in table S1. Stable gene knockdown cells were generated after lentiviral vector transduction and selection in medium containing puromycin (3 μg/ml; Sigma). Lentiviral particles were harvested by repeated freezing and thawing 3 times, and filtered by 0.45 μm filters. Naïve Huh7 cells were seeded into a muti-well plate and culture medium was discarded when cell confluence was approximately 80%, followed by twice 1×PBS washing. Harvested viruses were added and incubated at 37 ̊C with 5% CO_2_ for 48 hours. Then stable gene knockdown cells were selected and expanded by adding puromycin (3 μg/ml; Sigma). The KPNA1 or KPNA2 RNA level was quantified using a SYBR Green–based real-time PCR assay according to the manufacturer’s instructions. GAPDH was used as housekeeping gene to normalize gene expression using the 2-∆∆Ct method. The primer sequences were as follows: KPNA1 sense 5’- TTCCAAAAGCCCA GAGCAACAGC -3’; KPNA1 anti sense, 5’- CCACTACTCCTGGTGTGCTGAT -3’; KPNA2 sense, 5’-CTGTTGGCTCTCCTTGCAGTTC-3’; KPNA2 anti sense, 5’- GCAGGATTCTTGTTGCGGCAAAG-3’.

**Statistical Analysis.** Statistical analysis was performed using the non-paired, non-parametric test (Mann-Whitney test; GraphPad Prism software, GraphPad Software Inc., La Jolla, CA). All results were presented as mean ± standard errors of the means (SEM). P values <0.05 were considered as statistically significant.

**Supplementary table 1. shRNA sequences.**

| ID | Symbol | TargetTaxonld | TargetSeq | GeneDesc | OligoSeq |
| --- | --- | --- | --- | --- | --- |
| 1^#^ | KPNA1 | Human | GCAGTTATTCAAGCGGAGAAA | Karyopherin α1 (importin α5) | CCGGGCAGTTATTCAAGCGGAGAAACTCGAGTTTCTCCGCTTGAATAACTGCTTTTTG |
| 2^#^ | KPNA1 | Human | CCCTCCTATTGATGAAGTTAT | Karyopherin α1 (importin α5) | CCGGCCCTCCTATTGATGAAGTTATCTCGAGATAACTTCATCAATAGGAGGGTTTTTG |
| 3^#^ | KPNA1 | Human | GCCCTCTCATATCTATCAGAT | Karyopherin α1 (importin α5) | CCGGGCCCTCTCATATCTATCAGATCTCGAGATCTGATAGATATGAGAGGGCTTTTTG |
| 4^#^ | KPNA1 | Human | GCCTTTGATCTTATTGAGCAT | Karyopherin α1 (importin α5) | CCGGGCCTTTGATCTTATTGAGCATCTCGAGATGCTCAATAAGATCAAAGGCTTTTTG |
| 5^#^ | KPNA1 | Human | CTGCTGAATTTCGGACAAGAA | Karyopherin α1 (importin α5) | CCGGCTGCTGAATTTCGGACAAGAACTCGAGTTCTTGTCCGAAATTCAGCAGTTTTTG |
| 1^#^ | KPNA2 | Human | CGAATTGGCATGGTGGTGAAA | karyopherin α2 (RAG cohort 1, importin α1) | CCGGCGAATTGGCATGGTGGTGAAACTCGAGTTTCACCACCATGCCAATTCGTTTTTG |
| 2^#^ | KPNA2 | Human | CCTGGACACTTTCTAATCTTT | karyopherin α2 (RAG cohort 1, importin α1) | CCGGCCTGGACACTTTCTAATCTTTCTCGAGAAAGATTAGAAAGTGTCCAGGTTTTTG |
| 3^#^ | KPNA2 | Human | GCTGGTTTGATTCCGAAATTT | karyopherin α2 (RAG cohort 1, importin α1) | CCGGGCTGGTTTGATTCCGAAATTTCTCGAGAAATTTCGGAATCAAACCAGCTTTTTG |
| 4^#^ | KPNA2 | Human | GATGACATTGTCAAAGGCATA | karyopherin α2 (RAG cohort 1, importin α1) | CCGGGATGACATTGTCAAAGGCATACTCGAGTATGCCTTTGACAATGTCATCTTTTTG |
| 5^#^ | KPNA2 | Human | CTACCTCTGAAGGCTACACTT | karyopherin α2 (RAG cohort 1, importin α1) | CCGGCTACCTCTGAAGGCTACACTTCTCGAGAAGTGTAGCCTTCAGAGGTAGTTTTTG |

**
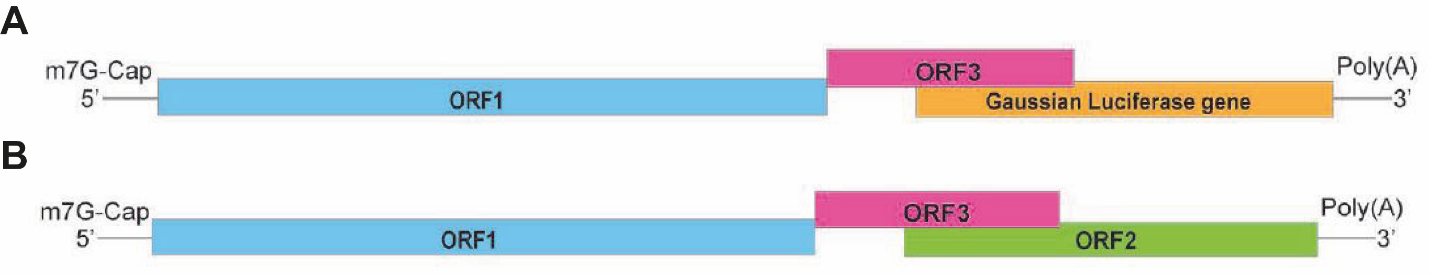
**

**Fig. S1. Diagram of HEV genome. (**A) HEV subgenomic replicon model (HEV-GT1-Luc or HEV-GT3-Luc), the open reading frame (ORF) 2 encoding the capsid protein was replaced by a Gaussia luciferase reporter gene for monitoring viral replication. (B) Full-length HEV genome (HEV-GT3-p6) for generating the infectious model.

**
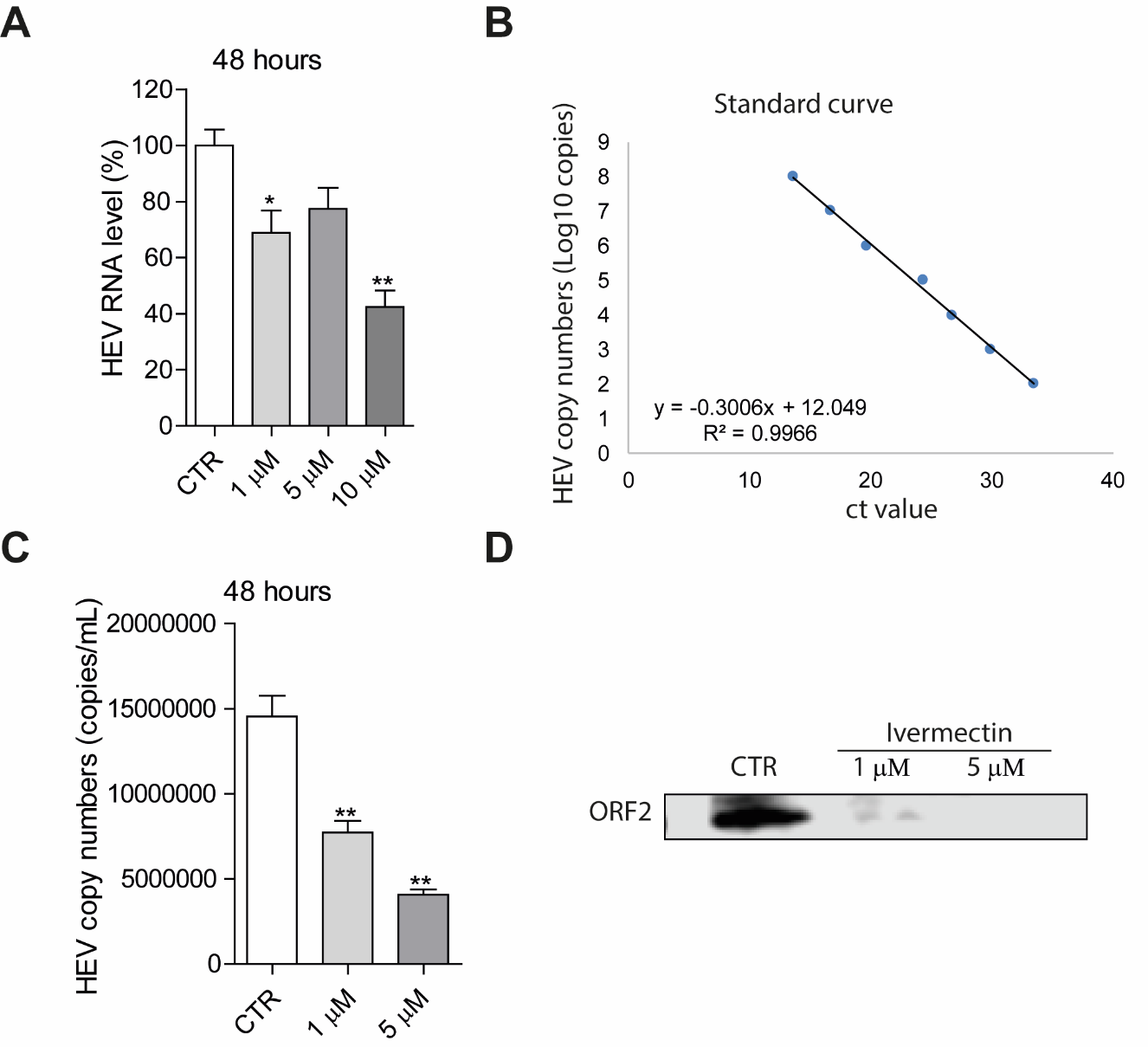
**

**Fig. S2. Anti-HEV activity of ivermectin.** (A) The infectious Huh7-p6 cell model was treated with indicated concentrations of ivermectin for 48 hours. The effects on viral RNA was quantified by qRT-PCR with primers targeting the RdRp region. The untreated group serves as control (CTR) (set as 100%) (n = 4 - 8). (B) qRT-PCR determined standard curve for calculating genome copy number. HEV plasmid based standard curve is generated by plotting the log copy number versus the cycle threshold (CT) value. (C) qRT-PCR analysis of HEV copy number in Huh7-p6 cells supernatant treated with ivermectin for 48 hours. The untreated group serves as CTR (n = 5 - 6). (D) Western blot analysis of HEV capsid protein level in Huh7-p6 cells supernatant. The untreated group serves as CTR.

**
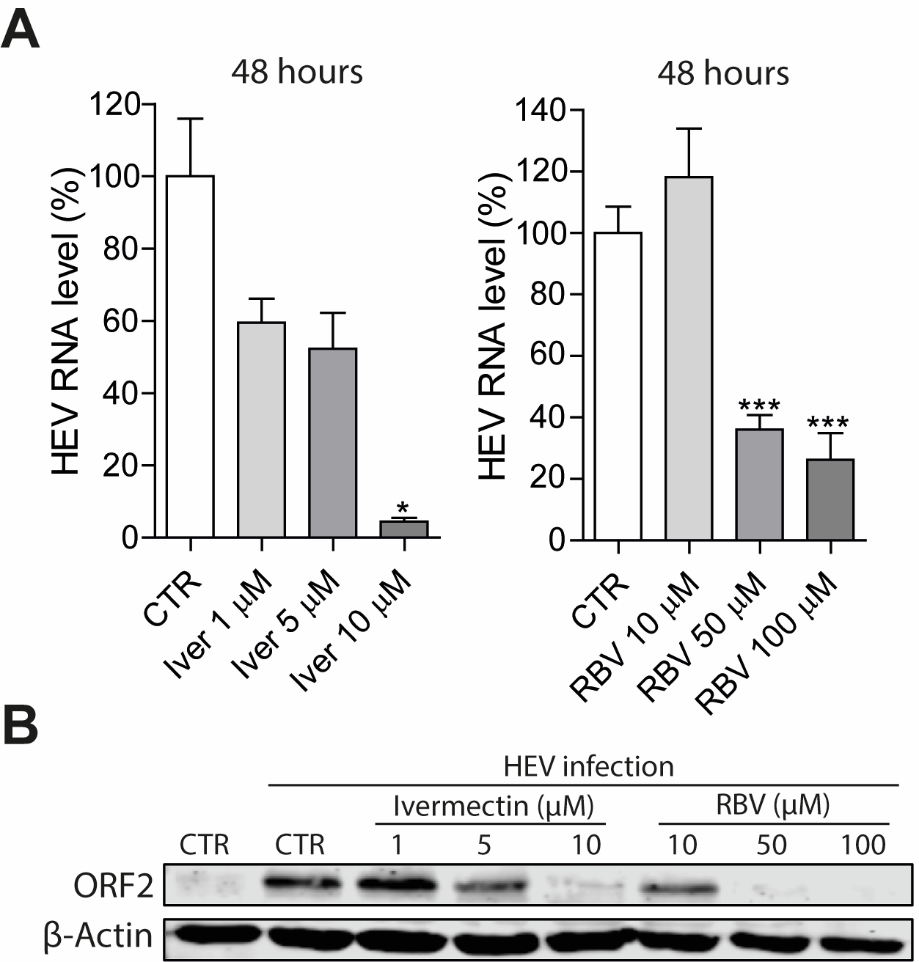
**

**Fig. S3. The effects of ivermectin treatment on the infectivity of produced HEV particles.** Naïve Huh7 cells were inoculated with HEV particles produced from HEV infected Huh7 cells 48 hours post-treatment with different concentrations of ivermectin or ribavirin (untreated set as control group). After the inoculation for 48 hours, the Huh7 cells were subjected to qRT-PCR analysis of HEV RNA (n = 4 - 8) (A), Western blotting analysis of HEV ORF2 protein (B). Data are presented as means ± SEM (*P < 0.05; **P < 0.01; ***P < 0.001).


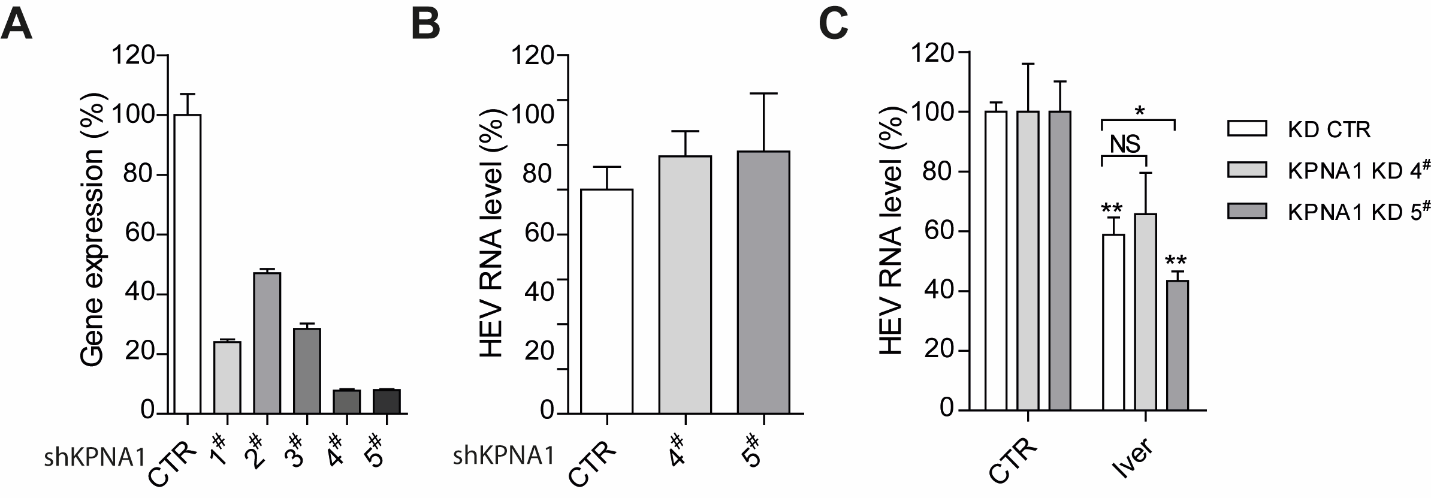


**Fig. S4. Gene silencing of KPNA1 by lentiviral RNAi did not affect HEV replication and abolish the anti-HEV activity of ivermectin.** (A) Knockdown of KPNA1 by lentiviral shRNA vectors. The effects on KPNA1 gene of 5 shKPNA2 clones were quantified by qRT-PCR (n = 4). The expression of KPNA2 in control vector transduced cells set as 100%. (B) The effects of KPNA1 knockdown on cellular HEV RNA in Huh7 cells harboring infectious p6 clone. Viral RNA was quantified by qRT-PCR (n = 4). (C) KPNA1 knockdown and control Huh7 cell lines harboring the infectious HEV model were treated with 5 μM ivermectin for 48 hours. The effects on viral RNA was quantified by qRT-PCR (n = 4 - 8). Data are presented as means ± SEM (*P < 0.05; **P < 0.01; ***P < 0.001).


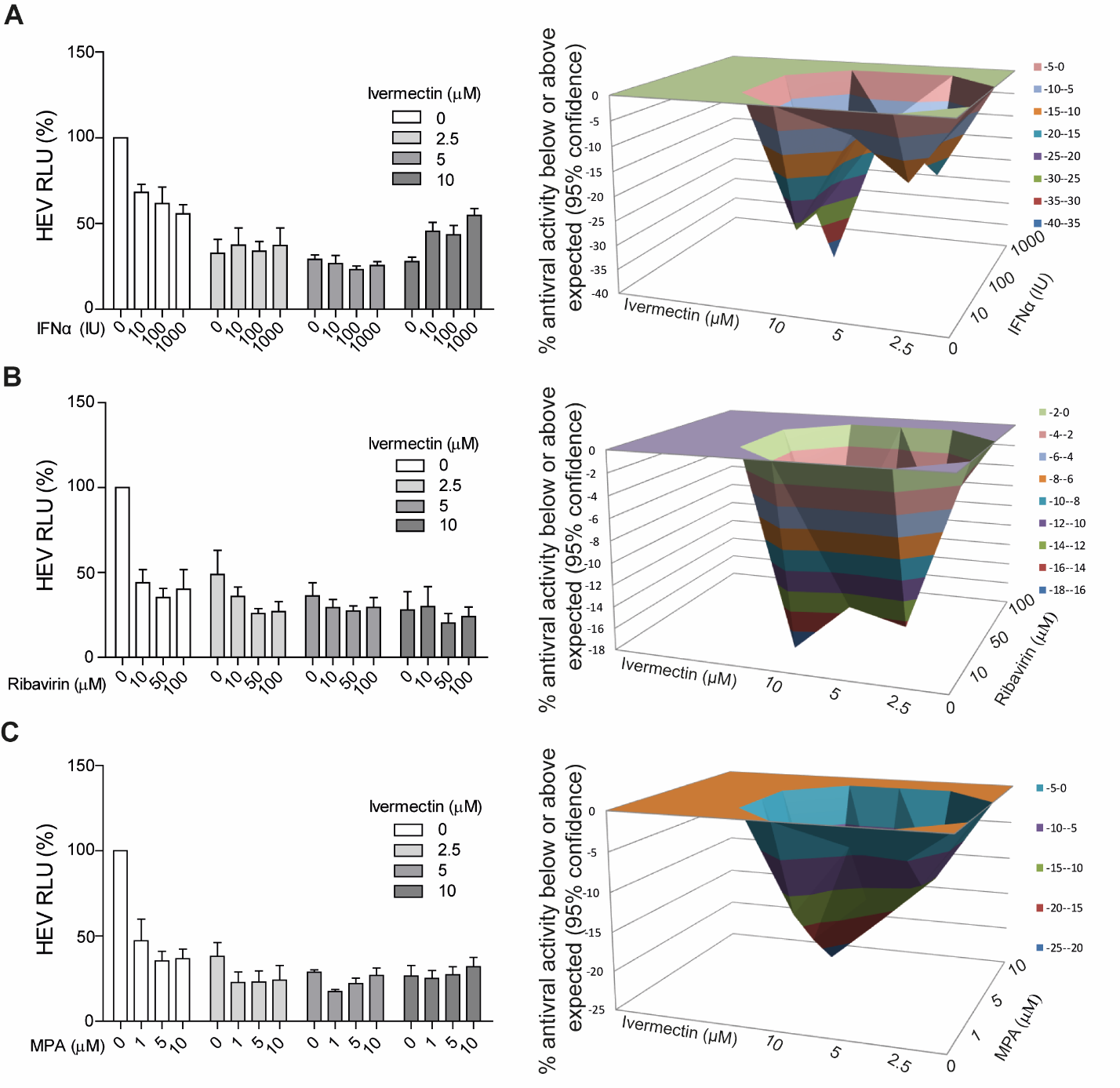


**Fig. S5. The combination of ivermectin with IFNα, ribavirin or MPA results in an antagonistic effect against HEV.** The antiviral effects of ivermectin in combination with interferon alpha (IFNα) (A), ribavirin (B) or mycophenolic acid (MPA) (C) were analyzed by the MacSynergyII model. The three-dimensional surface plot represents the differences (within 95% confidence interval) between actual experimental effects and theoretical additive effects of the combination at various concentrations (n = 4 - 5).

**Reference**

1. Shukla P, Nguyen HT, Faulk K, Mather K, Torian U, Engle RE, Emerson SU (2012) Adaptation of a genotype 3 hepatitis E virus to efficient growth in cell culture depends on an inserted human gene segment acquired by recombination. J Virol 86:5697-5707
